# International remittances, cash transfer assistance and voter turnout in Mexico

**DOI:** 10.1186/s40878-017-0065-z

**Published:** 2018-01-15

**Authors:** Ana Isabel López García

**Affiliations:** El Colegio de la Frontera Norte, Carretera escénica Tijuana - Ensenada, Km 18.5, San Antonio del Mar, 22560 Tijuana, Baja California Mexico

**Keywords:** International remittances, Political participation, Electoral turnout, Social programs, Poverty, Mexico

## Abstract

Most research on the political consequences of international migration conceptualizes financial remittances as being a substitute for state-provided assistance. This paper tests the actual validity of this assumption. Using data from the 2012–2016 Americas Barometer, the analysis confirms previous findings on the negative impact of financial remittances on electoral turnout intentions. However it reveals that this effect does not vary according to an individual’s beneficiary status of Conditional Cash Transfer (CCT) assistance. This finding is corroborated using data aggregated at the municipal level within Mexico. Accordingly, voter turnout rates in a given municipality for the 2012 presidential election are negatively associated with the percentage of households receiving remittances in that municipality. However, this association does not vary with the spending on CCT assistance within a given municipality. The evidence thus suggests that financial remittances undermine electoral participation through mechanisms other than the substitution of state-sponsored assistance, and as such further research is needed for us to discover what is really going on here.

## Introduction

Cash transfers can be delivered in the form of social assistance (by the state) or as remittances (by migrants). Existing studies demonstrate that both public and private transfers of money improve receivers’ well-being – by reducing poverty, vulnerability and other risks associated to financial shocks or unexpected expenses (Campos-Vazquez & Sobarzo, 2012, p. 9; López Córdova, 2006; Corona, 2014). Previous research also shows that cash transfers have important consequences for the political attitudes and behavior of receivers (De la O, 2013; Díaz-Cayeros, Magaloni, & Weingast, 2003; Díaz-Cayeros, Estévez, & Magaloni, 2009; Escribà-Folch, Meseguer, & Wright, 2015; Germano, 2013; Goodman & Hiskey, 2008; Layton & Smith, 2015; Pfütze, 2012). Yet, most studies on the political consequences of international migration conceptualize financial remittances as being a substitute for state-provided assistance. From this perspective, remittances decrease welfare demand and spending in migrant-sending countries (Abdih, Chami, Dagher, & Montiel, 2012; Ahmed, 2012, 2013; Doyle, 2015).

Recent evidence from Mexico reveals that it is not necessarily the case that financial remittances and cash transfer assistance target different segments of the domestic population. The lion’s share of remittance-receiving households are now concentrated in impoverished areas of the country, where state assistance in the form of Conditional Cash Transfers (CCT) have expanded significantly over the years (Banegas González & Escobar Latapí, 2012; Escobar Latapí et al., 2013; Cabello & Elton, 2017). By not considering this overlap between remittance recipients and cash assistance beneficiaries, scholars run the risk of drawing erroneous inferences about the actual mechanisms through which financial remittances affect electoral behavior in Mexico. This is a key issue, as the previous literature has emphasized that state-provided assistance and remittances can be interchanged from the recipient’s point of view.

Against this backdrop, this paper aims to contribute to the growing literature on the political effects of international migration by examining specifically the interactive effects of remittances and cash transfer assistance on electoral participation in Mexico. Although there are many types of state assistance programs, this paper focuses in particular on CCT ones. Both CCT assistance and financial remittances consist of money transfers; and both are delivered conditional on spending being made in specific ways, such as on food, healthcare, education, and the like. In light of this I ask in this paper: What are the joint electoral effects of international remittances and CCT assistance? Does the allocation and delivery of CCT assistance amplify or reduce the impact of international remittances on voter turnout?

Using data from the 2012–2016 Americas Barometer, the analysis confirms previous findings on the negative impact of financial remittances on electoral turnout intentions. However it reveals that this effect does not vary according to an individual’s beneficiary status vis-à-vis Oportunidades (Prospera), the largest anti-poverty CCT program in Mexico. This finding is corroborated using data aggregated at the municipal level. According to statistical models, turnout rates for the 2012 presidential election in a given municipality are negatively associated with the percentage of households receiving remittances in it. However, this association does not vary with the spending on this CCT program in a given municipality. These findings hold after controlling for a series of factors affecting electoral turnout at the individual and aggregate levels. The evidence thus suggests that financial remittances undermine electoral participation through mechanisms other than the substitution of state-provided assistance.

The paper is organized as follows. First, it provides background on international remittances and CCT assistance in Mexico. Second, it reviews the existing literature on the electoral consequences of international remittances and CCT assistance in the country. Based on this, it advances a series of hypotheses about the interactive effects of financial remittances and CCT assistance on voter turnout in Mexico. Third, it describes the quantitative data and methods that are used for testing these claims. Fourth, it presents and analyses the results obtained through a series of statistical models. Fifth, it discusses these findings and concludes by suggesting new avenues of research on how financial remittances shape electoral participation, not only in Mexico.

## The case of Mexico

Mexico is the world’s fourth-largest recipient of remittances (behind only India, China, and the Philippines), and the largest in Latin America (Yearbook of Migration & Remittances, 2016, pp. 124–127).^1^ Over 95% of the remittances to the country are sent from the United States (Yearbook of Migration & Remittances, 2016, pp. 131–132). Since 2005, family remittances to the country have exceeded USD 20 billion a year (Yearbook of Migration & Remittances, 2016, p. 130). International remittances constitute one of Mexico’s biggest sources of foreign income, accounting for approximately 2.5% of GDP in 2015 (Zong & Batalova, 2016; Campos-Vazquez & Sobarzo, 2012, p. 1; Li Ng, 2017). As of 2010, one in every 28 (1.3 million) Mexican households received international monetary transfers from abroad (CONAPO, 2010a; Yearbook of Migration & Remittances, 2016, p. 136). Mexican households use remittances to cover a range of basic needs, such as food, education, health, clothing and housing (Amuedo-Dorantes, Sainz, & Pozo, 2007; Valero-Gil, 2008; Yearbook of Migration & Remittances, 2016, p. 164).

As remittance inflows to Mexico have increased, government spending on welfare has expanded. Between 1997 and 2010 public social spending rose from 3.8 to 7.5% of the country's GDP (OECD, 2016). One of the social programs with the largest allocations in the national budget is Oportunidades (later renamed as Prospera). Oportunidades is the second largest CCT program in the whole of Latin America. The goal of this program is to improve the nutrition, health and education of poor children. Monetary grants are given to impoverished families so parents can afford sending their children to school and increase the quality of children’s food consumption. Beneficiaries of the program are chosen based on objective measures of poverty and assessed every 2 months; if they fail to meet the program’s education, nutrition, and health goals, cash transfers are withdrawn. This federal scheme started in 1994 by targeting poor rural households. Since 2000, however, poor households in both rural and urban areas benefit from the program.^2^ In 2000, 2.6 million families benefited from Oportunidades; by 2012, 6.5 million families were beneficiaries of this anti-poverty program. Nearly 7 million families (24.3 million people) are now beneficiaries of this program; of these, 68% live in rural areas of Mexico (“De Solidaridad a Prospera,” 2014). Between 1997 and 2014 spending  in Oportunidades rose from 0.01 to 0.4% of the country's GDP.  Also, the amount of the program's transfer increased by 60% in real terms, between 2001 and 2014 (Araujo & Suárez Buitrón, 2013, pp. 4-5; Dávila Lárraga, 2016, pp. 8-9).

Some accounts suggest that the Oportunidades program should nullify the emigration intentions of beneficiaries, since they must stay in the country to meet the program’s requirements (Stecklov, Winters, Stampini, & Davis, 2005). However recent studies demonstrate that this effect only holds true for the direct beneficiaries of the program (that is, women and the younger members of the household) (Angelucci, 2004, 2015; Azuara, 2009). For instance Angelucci (2015) uses a random sample from villages eligible for Oportunidades, and finds that labor emigration increases by 50% after a village receives the first transfers made as part of the program. Azuara (2009) also finds an increase in emigration rates from Mexican rural villages after the implementation of the Oportunidades program. These scholars attribute this effect to the impact that this CCT scheme has on households’ human capital and financial capacity for accessing loans. Although impoverished households face the greatest financial constraints to funding migration (Chiquiar & Hanson, 2005; McKenzie & Rapoport, 2010), these studies reveal that poor households in Mexico are nevertheless able to finance international emigration through safety net programs like Oportunidades (Angelucci, 2015).

In effect, since the mid-1990s, Mexican emigrants to the United States have been increasingly coming from the poorer federal states located in the center and south of the country—namely, Chiapas, Guerrero, Oaxaca, and Veracruz (CONAPO, 2010b). Presently, impoverished municipalities of Mexico have international emigration rates well above the national average (Escobar Latapí, 2012). Federal states with high poverty levels (such as Guerrero, Michoacán, Puebla, and Oaxaca) are also among the biggest recipients of financial remittances in the country (CONEVAL, 2015; Yearbook of Migration & Remittances, 2016, p. 133).^3^ Between 1992 and 2006 remittances to the poor in Mexico increased by 400% (Escobar Latapí, 2009, p. 89). As of 2014, 23.8 million people received international remittances and lived below the poverty line in Mexico (Cabello & Elton, 2017). An important share of remittance-receiving households in Mexico are now located in rural areas (44%) and in municipalities sitting below the poverty line (40%) (Yearbook of Migration & Remittances, 2016, p. 162), where the majority of beneficiaries of state assistance programs are clustered.

According to Mexico’s 2014 National Survey on Household Income and Expenditure (ENIGH), 49% of remittance receiving households in the country benefit from various forms of state-sponsored assistance, including: Oportunidades (Prospera), Procampo, Apoyo Alimenticio, Empleo Temporal, and other such social programs. By contrast, only 30% of the households that do not receive remittances from abroad benefit from state assistance— in the form of the Oportunidades or other such social programs. Approximately 31% of remittance-recipient households in Mexico are located in the first two income deciles. Within the first income decile, 58% of remittance-recipient households benefit from state-sponsored assistance; within the second income decile, 62% do so (Cabello & Elton, 2017). Similar trends are found by Banegas González and Escobar Latapí (2012) too. Using survey data from 2009, their analysis demonstrates that 51.3% of migrant households in Mexico’s poorest municipalities receive state assistance—in the form of the Oportunidades program. In sum, evidence from Mexico suggests that an important share of remittance recipients are actually beneficiaries of state assistance programs too, and especially CCT assistance. For these households financial remittances and CCT assistance are complements rather than substitutes.

## Existing research

However previous research in the context of Mexico has looked at the electoral effects of remittances and CCT assistance separately. On the one hand, existing studies demonstrate that international remittances have a negative effect on voter turnout in the country (Germano, 2013; Goodman & Hiskey, 2008). Analyzing aggregate data, Goodman and Hiskey (2008) find that participation rates for the 2000 presidential election in Mexico were negatively associated with the percentage of households receiving remittances at the municipal level. They corroborate this result with survey data, and find that those living in Mexican towns with high levels of emigration were less likely to participate in that election. This finding holds true regardless of whether the individuals in question were involved in nonpolitical community organizations or not. Similarly, using survey data collected in ten rural communities in Michoacán, Mexico, Germano (2013) shows that those dependent on international remittances were less likely to lobby local officials for financial assistance, and, therefore, were less likely to either reward or punish the incumbent party in the 2006 presidential election for poor economic performance.

This impact is attributed to recipients substituting these financial remittances for state-sponsored assistance. Since those who receive remittances are more capable of providing credit, education, healthcare, housing, infrastructure, and other public goods for themselves—as the argument goes—they are less likely to demand state assistance. Moreover, since they receive income from abroad, remittance recipients are less vulnerable to fluctuations in the national economy. As a result, they have fewer grievances against the government for poor economic performance and fewer incentives to reward (or punish) the incumbent party through electoral means. In sum, although international remittances increase the disposable income of recipients, which, according to resource models of political participation, should increase the likelihood of voting (Brady, Verba, & Scholzman, 1995), remittance-receiving individuals (or households) are less likely to go to the polls—because international remittances both compensate for poor economic performance at home and act as a substitute for state-provided assistance. This line of reasoning is consistent with the existing studies showing that international remittances are negatively related to welfare demand and spending in migrants’ home countries (Abdih et al., 2012; Ahmed, 2012, 2013; Doyle, 2015). Following this hypothesis, one could easily be led to believe that remittance-receiving individuals (and households) are less likely to benefit from state-sponsored assistance, including CCT programs. Recent evidence from Mexico casts doubt on these previous theoretical assumptions however.

On the other hand, various studies on Mexico show that CCT assistance does promote greater electoral turnout among its beneficiaries. For instance, using an experimental research design, De la O (2013) compares villages participating in the early and late stages of Oportunidades (formerly known as Progresa), and finds that early enrolment was associated with an increase of 7% in voter turnout and an increase of 16% in the vote share for the incumbent party (the Partido Revolucionario Institucional, PRI) in the 2000 presidential election. Similarly, using exit poll data from the newspaper *Reforma*, Díaz-Cayeros et al. (2009) report that in the 2006 presidential election Oportunidades beneficiaries were 11% more likely to vote for the presidential party candidate, Calderón, from the Partido Acción Nacional (PAN), and 7% less likely to vote for Andrés Manuel López Obrador, from the main opposition party the Partido de la Revolución Democrática (PRD).

In the analysis of Díaz-Cayeros et al. (2009, p. 234), CCT beneficiaries are seen as rational individuals who vote according to their own financial concerns. That is, they punish incumbents when economic conditions worsen and reward them when the reverse occurs. By providing beneficiaries with a safety net, so the argument goes, CCT assistance prevents politicians from losing political support—or being punished at the polls—for poor economic performance. Moreover these authors contend that improvements in one’s level of wellbeing can lead CCT beneficiaries to form attachments to the incumbent party and the state, and, consequently, they are more likely to go to the polls and reward them in return.

Other scholarly accounts argue that CCT programs, like Oportunidades, promote “issue voting” (Layton & Smith, 2015, p. 859). They raise awareness and knowledge about government efforts regarding redistributive spending among beneficiaries, and consequently increase the latter’s incentives to show up at the ballot box and reward or punish the incumbent party accordingly. By regularly attending public schools and health centers, CCT beneficiaries can also become more familiar with state bureaucracy—which can in turn lower over time the cognitive or psychological costs that beneficiaries associate with voting (Layton & Smith, 2015, p. 859). Besides, by increasing beneficiaries’ income, CCT programs offset these costs of showing up at the polls—including transportation costs to the polling station, taking time off from work, and even better children’s health and educational outcomes (De la O, 2013, p. 10; Layton & Smith, 2015, pp. 856–58).^4^ Another reason why CCT assistance can promote electoral turnout among its beneficiaries is related to effects on voter registration (De la O, 2013, p. 10; Layton & Smith, 2015, p. 859). To be enrolled in the program, recipients must have an official identity card. In Mexico, the most common form of identification is the voting card.

In sum, although CCT programs and financial remittances increase the income of recipients and both types are given upon conditions regarding spending, they have opposing effects on electoral participation, at least according to existing studies on Mexico. Financial remittances allegedly drive recipients away from the state and the electoral arena, whereas CCT assistance has the opposite effects. Contrary to the findings of the previous literature (Abdih et al., 2012; Ahmed, 2012, 2013; Doyle, 2015; Germano, 2013; Goodman & Hiskey, 2008), evidence from Mexico suggests that remittances and CCT assistance are not necessarily targeting different types of households (individuals). In light of this shortcoming in the existing literature, the key research question that now needs to be addressed is: In what ways (if any) does state assistance play a part in the effect that international remittances have on electoral turnout in Mexico (and beyond)? More specifically, what are the electoral effects of international remittances under CCT assistance?

Based on the assumption that remittances and state-provided assistance are (perfect) substitutes, we should expect that the effects of international remittances on voter turnout vary with the receipt of CCT assistance. In other words, receipt of remittances decreases the likelihood of a person voting—but this effect changes by the receipt of CCT assistance from the government. The following hypotheses can therefore be advanced:

- H1. *Remittance-recipients are less likely to vote than non-remittance recipients. However individuals who simultaneously receive remittances and CCT assistance are more likely to vote than those individuals who only receive international remittances.*
- H2. *International remittances are negatively related to electoral turnout rates in a given municipality. Yet the impact of international remittances on electoral turnout weakens with the spending in CCT assistance in that municipality.*

## Research design

To test the above claims, I use two approaches. The first one uses individual data, and tests whether receiving CCT assistance influences the intentions of remittance recipients to participate in presidential elections. The second approach uses data aggregated at the municipal level and tests whether spending as part of the Oportunidades program affects the impact of international remittances on turnout rates for the 2012 presidential election across different municipalities of the country.

### Individual-level data and methods

Individual-level data is drawn from the 2012–2016 waves of the Americas Barometer, carried out by the Latin American Public Opinion Project (LAPOP) at Vanderbilt University. I utilize this particular survey because it allows us to distinguish individuals’ electoral participation patterns and statuses as remittance recipients and CCT beneficiaries. Of all the Americas Barometer waves available since 2008, I only use those for 2012–2016. In previous waves of this survey, Mexican participants had only been asked whether themselves or their family members had received any monthly assistance from the state in the form of money or goods. From this question alone, it is difficult to identify what type of social assistance was received (whether it was in cash or kind), whether it was delivered with conditions or without conditions, to whom (who the beneficiaries are), and from what government level (municipal, state, or federal). Since 2012, however, Mexican respondents have been explicitly asked whether they are beneficiaries of the Oportunidades (Prospera) program or not.

The main dependent variable is electoral turnout intentions. It is a binary variable, coded 1 if respondents said they would vote, if the next presidential election was being held this week, and 0 otherwise.

The main independent variables are: individuals’ status as remittance receiver and as CCT assistance beneficiaries. Remittance-recipient status is registered as 1 if respondents answered affirmatively to the question “Do you, or someone in your household, receive money from abroad?” and 0 otherwise. Meanwhile, the status of being a beneficiary of CCT is coded as 1 if respondents answered affirmatively to the question “Do you, or someone in your household, receive monthly assistance as part of the Oportunidades (Prospera) program?” and 0 otherwise.

The statistical models account for other factors that, according to previous research, affect the likelihood of an individual showing up at the polls. These include: previous voter turnout, partisanship, support for democracy, trust in elections, political interest, social membership, and crime victimization.^5^ Also considered are other variables that might affect electoral turnout intentions, such as the respondent’s age,^6^ gender,^7^ educational attainment level, household income,^8^ employment status,^9^ the size of their place of residence,^10^ positive assessments or not of the economic situation of the country, and intentions to live abroad.^11^ The coding of these control variables is described in the Appendix. To capture unobserved heterogeneity that varies across waves of the LAPOP survey, also included are year dummies.

The estimation strategy consists of a series of linear probability models. One concern, however, is that individuals who receive remittances are not randomly selected. In correcting for this, I employ a matching procedure, which consists of pairing individuals with similar characteristics into two groups—with one group receiving the treatment, and the other not. In this study, the treatment group is the one that receives remittances. Pretreatment variables are respondents’ work situation, income earned through labor (or pensions), size of place of residence, and household size. This is because remittance recipients are more likely to: be unemployed or lack a stable labor income, be low-income earners themselves, reside in rural areas of the country, and live in households with various dependents. By adjusting for the distribution of covariates between remittance recipients and nonrecipients, matching can allow us to separate the effect of remittances from other factors shaping individuals’ electoral turnout intentions—and thus to create more valid comparisons. Individuals who receive CCTs are also likely to be non-randomly selected. However the pretreatment variables used for matching can allow us to correct for the non-random selection of CCT recipients as well, since they also tend to lack a stable income, be low-income earners, reside in rural areas, or live with other dependent relatives.

To match respondents, I use the exact matching procedure, which matches observations together if they are identical on a set of covariates. Those observations that are not matched are discarded.^12^ Although complete data is available for 2431 individuals, the number of observations is reduced to 734 after matching. Of these, 130 observations belong to the treatment group. Since many observations are lost after matching, the statistical power of the models on the basis of using individual data might be low.

### Aggregate-level data and methods

As a robustness check, I re-run the analysis using aggregate data at the municipal level. Aggregate data was compiled for all of the Mexican municipalities that participated in the 2012 presidential election. However complete data was available for 2443 such municipalities. In 2012 Mexico had 2456 municipalities—in other words, 13 are unaccounted for. The aggregate data used in the analysis comes from a variety of sources. Information on electoral participation rates was collected from the National Electoral Institute of Mexico (INE). Data on international remittances comes from the Mexican National Population Council (CONAPO), and is based on the 2010 Mexican census conducted by the Mexican National Institute of Statistics and Geography (INEGI). Information on CCT assistance and the socioeconomic and demographic characteristics of municipalities was collected from INEGI and the National Council for the Evaluation of Social Development Policy (CONEVAL, 2015).

The dependent variable is turnout in the 2012 presidential election, whereas the main dependent variables are the proportion of households receiving remittances and the spending in Oportunidades per 1000 habitants in a given municipality.^13^ Also included are other relevant socioeconomic and demographic characteristics that can affect voter turnout rates at the municipal level. See the Appendix for a description of the variables used in models based on data aggregated at the municipal level. The models also include state dummies to capture otherwise unobserved heterogeneity that varies across Mexican federal states.

The analysis based on aggregate data is limited to the cross-sectional level. This is because the relevant municipal-level data on international migration indicators as well as socioeconomic ones is only gathered every 10 years. Also, since the proportion of households receiving remittances in a given municipality and the CCT spending allocated to a particular municipality are not randomly assigned, there is a risk that model estimates could be biased. To address this, instrumental variables are used to enable the prediction of the random assignment of these two variables. These are: the proportion of households that received remittances in a given municipality in 2000, and the number of people aged under 15 per 1000 habitants in a given municipality.

All estimations were conducted using R. To correct for further heterocedasticity, robust standard errors are used in all of the models.

## Results

Table 1 shows the summary statistics for the variables included in the models based on data collected at the individual level. As seen, approximately 85% of respondents said they would indeed vote if presidential elections were to be held this week; 7% receive international remittances; while 22% are CCT beneficiaries. However only 2% of those surveyed simultaneously benefit from international remittances and CCT assistance.

**Table 1:** Descriptive Statistics. Americas Barometer 2012–2016

Statistic	N	Mean	St. Dev.	Min	Pctl(25)	Median	Pctl(75)	Max
Turnout intentions	2431	0.85	0.36	0	1	1	1	1
Remittance recipient	2431	0.07	0.25	0	0	0	0	1
CCT beneficiary	2431	0.22	0.41	0	0	0	0	1
Remittance and CCT beneficiary	2431	0.02	0.15	0	0	0	0	1
Trust in elections	2431	2.35	1.89	0	0	2	4	6
Support for democracy	2431	3.74	1.73	0	3	4	5	6
Previously voted	2431	0.71	0.45	0	0	1	1	1
Party sympathizer	2431	0.28	0.45	0	0	0	1	1
Political interest	2431	1.18	0.94	0	0	1	2	3
Community participation	2431	2.63	2.16	0	1	3	4	12
Crime victimization	2431	0.63	1.70	0	0	0	1	20
Emigration intentions	2431	0.16	0.37	0	0	0	0	1
Perceptions of the national economic situation	2431	0.34	0.55	0	0	0	1	2
Employed	2431	0.56	0.50	0	0	1	1	1
Labor income	2431	5.42	4.91	0	1	5	9	16
Size of place of residence	2431	2.44	1.14	0	2	3	3	4
Female	2431	0.48	0.50	0	0	0	1	1
Age (years)	2431	39.67	15.51	18	27	37	50	93
Educational attainment (years)	2431	9.43	4.21	0	6	9	12	18
Household income	2431	4.89	4.84	0	1	3	9	16
Household size	2272	4.51	2.08	1	3	4	5	18
2012 wave	2431	0.20	0.40	0	0	0	0	1
2014 wave	2431	0.37	0.48	0	0	0	1	1
2016 wave	2431	0.43	0.50	0	0	0	1	1

Table 2 shows the summary statistics for the variables included in the models using aggregate data collected at the municipal level. As seen, the average voter turnout for the 2012 presidential election was 64.7% (range: 0%–94.5%). Between 2005 and 2010 the average share of households (for all municipalities) receiving remittances was 6.52% (range: 0%–48.7%). In 2012 the average spending per 1000 habitants in the Oportunidades program was 1088.95 thousand Mexican pesos.

**Table 2:** Descriptive Statistics. Aggregate Data at the Municipal Level

Statistic	N	Mean	St. Dev.	Min	Pctl(25)	Median	Pctl(75)	Max
Remittance receiving households (%)	2456	6.50	7.25	0.00	1.47	3.72	8.99	48.70
Turnout in the 2012 election (%)	2446	64.71	10.71	0.00	58.58	64.87	70.82	94.48
Remittance receiving households (2000)	2443	6.52	7.70	0.00	1.00	3.42	9.46	53.71
CCT spending (per 1000 habitants)	2456	1088.95	748.52	0.00	533.14	1062.59	1581.36	20,906.55
Social marginalization (index)	2456	0.00	1.00	−1.89	−0.79	−0.14	0.63	4.44
High and very high levels of marginalization (binary)	2456	0.25	0.43	0	0	0	0	1
Population size	2456	45,694.50	132,385.2	93	4264.5	12,730.5	32,664.2	1,800,000
Population density	2452	280.18	1178.54	0.14	18.69	52.07	133.34	17,423.40
Population under 15 (per 1000 habitants)	2456	310.46	52.95	136.23	275.15	305.47	341.22	553.74
Sex ratio	2456	95.59	6.57	63.20	91.80	95.40	99.30	142.00
Homicide rate (per 1000 habitants)	2456	0.10	0.22	0.00	0.00	0.02	0.11	2.92

Table 3 shows the estimates obtained from a series of linear probability models, using data collected at the individual level. For comparison purposes, the estimates obtained both before and after matching are displayed herein. As seen, the results are fairly consistent across these two methods. Therefore I only report the estimates obtained after matching (Models 1 and 2). As seen in Model 1 (in Table 3), remittance recipients are less likely to participate in presidential elections than nonrecipients are. This is consistent with the existing studies that show that there is negative relationship between remittances and electoral turnout (Goodman & Hiskey, 2008; Germano, 2013). However the models reveal that CCT assistance does not affect the probability of electoral turnout. This contradicts the results obtained by De la O (2013) and Layton and Smith (2015) on the electoral consequences of CCT asssistance.

**Table 3:** Linear probability models

	Dependent variable: Turnout intentions			
	Post-matching		Pre-matching	
	(1)	(2)	(3)	(4)
CCT beneficiary	0.032	0.050	0.014	0.015
	(0.035)	(0.040)	(0.017)	(0.018)
Remittance recipient	−0.077^**^	−0.056	−0.051^*^	−0.046
	(0.037)	(0.046)	(0.029)	(0.035)
CCT^*^remittances		−0.070		−0.015
		(0.083)		(0.062)
Trust in elections	0.019^***^	0.019^**^	0.015^***^	0.015^***^
	(0.007)	(0.007)	(0.004)	(0.004)
Support in democracy	0.020^***^	0.021^***^	0.014^***^	0.014^***^
	(0.009)	(0.009)	(0.004)	(0.004)
Previously voted	0.132^***^	0.132^***^	0.170^***^	0.170^***^
	(0.043)	(0.043)	(0.020)	(0.020)
Party sympathizer	0.126^***^	0.126^***^	0.102^***^	0.102^***^
	(0.032)	(0.032)	(0.012)	(0.012)
Interest in politics	0.047^***^	0.048^***^	0.060^***^	0.060^***^
	(0.018)	(0.018)	(0.008)	(0.008)
Community participation	−0.002	−0.002	0.004	0.004
	(0.008)	(0.008)	(0.003)	(0.003)
Crime victimization	0.0005	−0.0005	0.005	0.005
	(0.015)	(0.015)	(0.004)	(0.004)
Perceptions of national economy	0.005	0.007	−0.004	−0.003
	(0.027)	(0.027)	(0.013)	(0.013)
Emigration intentions	0.00002	0.001	−0.019	−0.019
	(0.040)	(0.040)	(0.021)	(0.021)
Educational attainment	0.007^*^	0.007^*^	0.004^*^	0.004^*^
	(0.005)	(0.005)	(0.002)	(0.002)
Household income	−0.011^***^	−0.011^***^	−0.005^*^	−0.005^*^
	(0.005)	(0.005)	(0.002)	(0.002)
Size of place of residence	0.015	0.016	0.002	0.002
	(0.015)	(0.016)	(0.006)	(0.006)
Employed	−0.034	−0.034	−0.019	−0.019
	(0.030)	(0.030)	(0.015)	(0.015)
Female	0.012	0.011	−0.005	−0.005
	(0.032)	(0.032)	(0.015)	(0.015)
Age	−0.006	−0.006	−0.006^**^	−0.006^**^
	(0.005)	(0.005)	(0.003)	(0.003)
Age (squared)	0.0001	0.0001	0.0001^**^	0.0001^**^
	(0.0001)	(0.0001)	(0.00003)	(0.00003)
2012 wave	0.013	0.015	0.009	0.009
	(0.048)	(0.048)	(0.025)	(0.025)
2014 wave	0.039	0.040	0.028	0.028
	(0.051)	(0.051)	(0.025)	(0.025)
Constant	0.629^***^	0.616^***^	0.624^***^	0.623^***^
	(0.140)	(0.143)	(0.061)	(0.061)
Observations	734	734	2419	2419
R^2^	0.145	0.146	0.151	0.151
Adjusted R^2^	0.121	0.121	0.144	0.144
Residual Std. Error	0.319 (df = 713)	0.319 (df = 712)	0.332 (df = 2398)	0.332 (df = 2397)
F Statistic	6.056^***^ (df = 20; 713)	5.816^***^ (df = 21; 712)	21.330^***^ (df = 20; 2398)	20.309^***^ (df = 21; 2397)

Notes: Coefficients are statistically significant at ^*^p < 0.1; ^**^p < 0.05; ^***^p < 0.01. Robust standard errors are in parentheses

To reiterate, the aim of this paper is to examine whether the relationship between international remittances and voter turnout changes with the delivery of CCT assistance. Model 2 includes an interaction term, which estimates the additional effects of CCT assistance on electoral turnout intentions among individuals receiving international remittances. As seen in Model 2 (in Table 3), the interaction term does not pass the threshold of statistical significance. This means that receiving cash assistance from the state does not affect the probability of those individuals who receive remittances voting. That is, remittance recipients are less likely to vote—regardless of whether they benefit from money transfers from the state or not.

Additionally, Models 1 and 2 (in Table 3) tell us that the probability of voting is higher among those individuals who have previously voted, identify with a political party, show more interest in politics, have greater confidence in the electoral process, and exhibit greater support for democratic politics. With regard to other control variables it can be seen that more educated respondents are more likely to vote, whereas those living in low-income households are less inclined to show up at the polls.

To further investigate the interactive effects of CCT assistance and international remittances on voter turnout, I specify a series of models using data aggregated at the municipal level. Table 4 reports the coefficients obtained using Ordinary Least Squares (OLS), and a two-stage least squares (2SLS) estimation. However I only report estimates obtained using 2SLS (Models 5 and 6). As seen in Model 5 (in Table 4), the share of households that receive international remittances is negatively related to the rate of turnout for the 2012 presidential election in a given municipality. More precisely, for every ten-unit increase in the proportion of households receiving remittances in a given municipality the turnout rate there decreases by 5 points. This finding reinforces the idea that international remittances have a negative effect on electoral turnout (Goodman & Hiskey, 2008; Germano, 2013). However it can also be seen that the spending as part of the Oportunidades program in a given municipality is not statistically related to voter turnout rates. This again confirms the finding that CCT assistance has no meaningful effect on electoral participation (Cornelius, 2004; Imai, King, & Velasco Rivera, 2016).

**Table 4:** Linear regression models

	Dependent variable: Turnout in the 2012 presidential election			
	Instrumental variables		OLS	
	(5)	(6)	(7)	(8)
Remittance receiving households	−0.498^***^	−0.517^**^	−0.414^***^	−0.211^***^
	(0.057)	(0.243)	(0.028)	(0.060)
Oportunidades spending	0.0003	0.0001	0.001^***^	0.002^***^
	(0.004)	(0.005)	(0.001)	(0.001)
Oportunidades^*^ Remittances		0.00002		−0.0002^***^
		(0.0002)		(0.0001)
Population density (log)	−0.911	−0.926	−0.515	−0.493
	(0.792)	(0.883)	(0.397)	(0.396)
Population size (log)	−4.045^***^	−4.070^***^	−3.818^***^	−3.672^***^
	(0.688)	(0.849)	(0.373)	(0.373)
Social marginalization (index)	−0.845	−0.793	−1.197^***^	−1.264^***^
	(1.856)	(2.176)	(0.463)	(0.475)
High and very high levels of marginalization	−0.120	−0.115	−0.004	−0.037
	(0.633)	(0.646)	(0.625)	(0.624)
Homicide rate	−1.695^**^	−1.722^**^	−1.667^**^	−1.456^**^
	(0.872)	(0.993)	(0.814)	(0.808)
Sex ratio	0.090^**^	0.090^**^	0.127^***^	0.124^***^
	(0.041)	(0.041)	(0.037)	(0.036)
Constant	75.947^***^	76.302^***^	68.965^***^	67.097^***^
	(11.264)	(13.522)	(4.442)	(4.480)
Observations	2432	2432	2443	2443
State effects	YES	YES	YES	YES
R^2^	0.572	0.571	0.576	0.578
Adjusted R^2^	0.565	0.564	0.569	0.571
Residual Std. Error	7.069 (df = 2392)	7.079 (df = 2391)	7.032 (df = 2403)	7.010 (df = 2402)
F Statistic			83.554^***^ (df = 39; 2403)	82.380^***^ (df = 40; 2402)

Notes: Coefficients are statistically significant at ^*^p < 0.1; ^**^p < 0.05; ^***^p < 0.01. Robust standard errors are in parentheses

To examine whether the relationship between remittances and voter turnout varies in the context of the delivery of the Oportunidades program, Model 6 (in Table 4) includes an interaction term between the spending on Oportunidades and the proportion of remittance-recipient households in a given municipality. However, the interaction term is not statistically significant. In other words, the effect of international remittances on voter turnout rates in a given municipality does not vary according to the spending as part of the Oportunidades program at the municipal level. These results are consistent with those obtained using data collected at the individual level.

It should be noted that, in the specifications based on data aggregated at the municipal level, the instrumental variables used pass the “weak instruments” and “Wu-Hausman” tests. These assess the strength of the instruments, and the consistency of the 2SLS estimation as compared to OLS respectively.^14^

### Discussion of results

Overall, the statistical analysis confirms that international remittances do indeed undermine voter turnout in Mexico. Analogous results were obtained by Goodman and Hiskey (2008) for the 2000 Mexican presidential election and by Germano (2013) for the same such election in 2006. This paper adds to and reinforces the literature by obtaining similar results, despite examining data that is quite distinct from the settings of previous studies. However the findings uncovered here do not support existing studies regarding the positive effects of CCT assistance on turnout (De la O, 2013; Layton & Smith, 2015). The analysis shows that electoral turnout is in fact not affected by CCT assistance provided via the Oportunidades program (Cornelius, 2004; Imai et al., 2016). Moreover, against the initial hypothesis of the study, the analysis reveals that the negative impact of international remittances on electoral turnout does not change with the delivery of cash transfers by the state. These results are robust when using data at both the individual and aggregate levels.

Hence the evidence suggests that international remittances have a negative effect on voter turnout in Mexico, irrespective of whether the recipients also benefit from cash transfers from the government. The findings thus suggest that the mechanisms through which international remittances influence voter turnout are other than by the substitution of state-provided assistance for remittances. This is an important contribution to the literature on the political consequences of international migration in sending countries, which has commonly attributed the negative effects of international remittances on voter turnout to the substitution of international remittances for state-sponsored assistance. In light of these new insights, the question that researchers on the political economy of remittances should now look at is: Why are international remittance recipients still less likely to vote even when benefiting from cash assistance from the state?

One possible explanation for these as yet unexplained discrepancies is that the amount of money that CCT beneficiaries receive is not in itself sufficient to satisfy a household’s needs—and therefore its electoral impact is ultimately negligible. In effect, even though CCT assistance has succeeded in reducing the number of people living in extreme poverty in Mexico it has simultaneously been ineffective in reducing the overall number of people living below the national poverty line (Montalvo, 2014). As of 2013, every CCT beneficiary family received, on average, a monthly transfer of 130 US dollars (IADB, 2013), whereas the average remittance sent to Mexico was 292.4 US dollars (Yearbook of Migration & Remittances 2016, p. 130). This is consistent with previous studies showing that remittances have a larger effect on well-being than cash transfer assistance (Waidler, Hagen-Zanker, Gassmann, & Siegel, 2017, p. 357; Hagen-Zanker & Leon Himmelstine, 2016).

Another explanation relates to the social remittances that often accompany the financial ones sent by migrants living in advanced democratic countries. Social remittances are defined as “the ideas, behaviors, identities, and social capital that flow from sending to receiving country communities,” being learnt and absorbed by migrants while living abroad—and intentionally and unintentionally transmitted to nonmigrants in their native country (Levitt, 1998, p. 926). Through the social remittances that they send home, migrants arguably have the potential to affect the political attitudes and practices of nonmigrants—and, therefore, to promote political transformation in their country of origin (Pérez-Armendáriz & Crow, 2010; Pérez-Armendariz, 2014; Córdova & Hiskey, 2015). Following this hypothesis, recipients of financial remittances who communicate frequently with relatives living abroad might perceive the delivery of CCT assistance as a right, not a privilege, and therefore as something that the government does not need to be rewarded for via elections. This is indeed plausible, considering that in Mexico electoral participation has long been induced through the clientelist disbursement of state assistance and welfare.

Oportunidades (Prospera) benefits cannot be rescinded due to recipients’ political orientation or activities. Also, the program itself is safeguarded against political manipulation by a ruling that prevents its coverage being extended during a federal election year. However, in the Mexican context it is likely that remittance recipients might consequently see the delivery of CCT (and other types of social assistance) as vote buying—and thus they prefer not to participate in elections (Luccisano & Macdonald, 2012). This reasoning is in line with previous studies showing that financial remittances reduce the incentives for recipients to engage in clientelist practices with state actors, and as part of that to vote for the incumbent party (Díaz-Cayeros et al., 2003; Pfütze, 2012; Escribà-Folch et al., 2015). However these accounts still assume that the financial remittances sent by migrants are used for substituting goods provided by political actors or the state. Although remittances can dampen the ability of political actors to buy electoral support, the electoral consequences of international remittances might actually be due to other mechanisms, as argued above. More research is needed to understand the channels through which financial remittances affect electoral participation in Mexico (and beyond).

## Conclusions

Most scholarship on the political consequences of international migration assumes that financial remittances reduce the demand for state-sponsored assistance. This paper provides evidence to the contrary, by drawing specifically on the case of Mexico. It shows that a growing share of international remittance recipients are simultaneously benefiting from cash transfers made by the Mexican government. This paper confirms previous scholarly findings on the negative effect of remittances on electoral turnout (Goodman & Hiskey, 2008; Germano, 2013). However its findings do not support the idea that financial remittances undermine voter turnout due to the substitution effect they have on the demand for (or provision of) state-sponsored assistance. What is shown is that the effect of international remittances on electoral turnout does not change with the delivery of CCT assistance.

In other words, international remittances lower electoral turnout rates regardless of whether recipients or municipalities benefit from cash  transfers from the state or not. This result casts doubt on the substitution effect that informs most literature on the electoral effects of international remittances. Of course, more research is needed to uncover the specific mechanisms through which financial remittances reduce recipients’ incentives to participate in elections in migrant-sending countries. However the evidence presented here should encourage scholars to look beyond the income effects of financial remittances, and instead pay more attention to the role of social and political remittances in helping (re)shape the attitudes and behavior of recipients toward politics—and indeed the state in general (Pérez-Armendáriz & Crow, 2010; Córdova & Hiskey, 2015; Meseguer, Lavezzolo, & Aparicio, 2016).

Clearly this study has its limitations. Scholars should corroborate the findings presented herein using different estimation strategies and alternative data that allows for distinguishing the remittance behavior of emigrants, the spending patterns of receivers, the extent to which remittances and cash assistance are fungible, and the electoral participation patterns of receivers. In-depth qualitative evidence and ethnographic accounts could shed further light on the joint electoral effects of remittances and CCT assistance. This study also focuses on only one social intervention, the Oportunidades program, and therefore only on the population segment benefiting from this type of CCT assistance—that is, the poor with young children. Further studies should examine whether these findings hold true when considering other sections of the population, and other types of state assistance, such as noncontributory pensions, health insurance schemes and unemployment benefits. In this regard, an interesting line of research would be to examine the interactive effects of subsidies to consumption and international remittances on electoral participation.

Another limitation of this study is that it considers only one type of political participation: voting. Further research should explore the ways in which international remittances interact with CCT programs, or other forms of state-sponsored assistance, and affect other types of political participation—such as protests, lobbying, or town hall meetings (Burgess, 2012). Although this study is based exclusively on the case of Mexico, the analysis could be extended to other migrant-sending countries in Latin America and Africa, where remittances have been related to political participation (Ebeke & Yogo Urbain, 2013; Dionne, Inman, & Montinola, 2014; Maydom, 2017), and CCT programs have become very popular over the past decades. The results of these future undertakings will allow us to better comprehend the exact mechanisms through which international remittances affect political participation not only in Mexico but elsewhere across the globe too.

## Appendix

### Individual data

Control variables

*Previously voted* is a dichotomous variable, measured as 1 if the participant reported having voted in past presidential elections and 0 otherwise.

*Party sympathizer* is a binary variable that takes the value of 1 if the respondent reports currently sympathizing with any political party and 0 otherwise.

*Support for democracy* is based on whether respondents agree with the claim that “democracy is the best form of government.” This variable is measured on a seven-point ordinal scale ranging from 0 to 6, where 0 indicates total disagreement with the statement.

*Trust in elections* is an ordinal variable that ranges from 0 to 6, with higher values indicating more trust in the electoral process.

*Political interest* is based on how much interest respondents have in politics. It is an ordinal variable that ranges from 0 to 3, with higher values indicating greater interest in politics.

*Community participation* is an additive index of four questions, asking respondents how often they attend meetings organized by religious groups, parents’ associations, neighborhood organizations, and women’s groups. It is an ordinal variable that ranges from 0 to 12, with higher values indicating higher attendance rates.

*Crime victimization* is measured as the number of times that the respondent has been victim of a crime in the last 12 months. It is included because Mexico experienced an escalation in drug-related violence during the period under study.

*Age* is the number of years old that the respondent is.

*Female* is a binary variable, coded 1 if the responded is female and 0 otherwise.

*Educational attainment* is measured as the number of years of schooling.

*Size of place of residence* is included to distinguish between rural and urban areas. It is measured as an ordinal variable, ranging from 0 to 4. Higher values indicate a greater population density.

*Household income* is measured in income quantiles (ranging from 0 to 16). It is the income that the household receives, including unearned labor income, such as money transfers.

*Household size* is measured as the number of people living in the respondent’s household.

*Labor income* is measured in income quantiles (ranging from 0 to 16). It is the income that the respondent earns through labor or pensions.

*Employed* is a binary variable, coded 1 if the respondent regularly earns an income through labor or a pension, and 0 otherwise.

*Perceptions of the economic situation of the country* is an ordinal variable that ranges from 0 to 2, with higher values indicating a better assessment thereof over the past 12 months.

*Emigration intentions* is a binary variable, coded 1 if the respondent intends to emigrate in the next 3 years, and 0 otherwise.

### Aggregate data

*Dependent Variables*

*Voter turnout* measures the total number of votes cast in the 2012 presidential election, divided by the total number of registered voters. It is a continuous variable that ranges from 0 to 100. Only votes cast by nationals who resided in the country at the time of the presidential election are considered.^15^

*Independent Variables*

*Remittance-recipient households* measures the share of households in a given municipality that reported having received remittances from abroad during the 2005–2010 period.

*CCT spending* measures spending per 1000 habitants in the Oportunidades program in thousands of Mexican pesos for the year 2012.

*Instruments*

*Remittances (2000)* measures the proportion of households in a given municipality that received remittances from abroad in the year 2000. Theoretically in 2012 the share of remittances will be larger in those municipalities where a large number of households received remittances in 2000. Using the past value of the proportion of households receiving remittances is an appropriate instrumental variable, because (conditional on all regressors) unobserved shocks that can affect electoral participation in the present are likely to be unrelated to the share of households that received remittances more than a decade ago (the exclusion restriction), while share of households that received remittances a decade ago predicts share of households that receives remittances now (the inclusion restriction).

*Population aged under 15* is registered as the number of inhabitants aged under 15 years old per 1000 persons in a given municipality. This variable predicts the spending of CCT assistance in a given municipality, since this type of assistance is delivered to support the health, food and education expenses of young children. But, given that this variable does not consider the age structure of the voting population, it does not affect electoral turnout rates. Therefore it is an appropriate instrument.

*Control Variables*

*Population size* measures the number of inhabitants in each municipality. It is included because voters are supposedly instrumentally rational, which means that the probability that an individual’s vote will be decisive decreases as population size increases (Geys, 2006b, pp. 642–643). Given the wide variation in population size across the municipalities included in the sample, the population size logarithm is thus included in the regression models.

*Population density* is used to distinguish between rural and urban areas. It divides the total size of the population by the area of the municipality, measured in square kilometers. The logarithm of this variable is included in the regression models.

*Social Marginalization Index*, as developed by CONEVAL, is the weighted mean of a series of normalized indices that measure the following dimensions of development: access to social security, access to health services, educational attainment level, access to food, access to housing, access to basic services, and the quality of one’s living space. A lower index score indicates a lower level of marginalization.

*Rate of homicide* measures the number of homicides per 1000 habitants that occurred in the 6 months prior to the 2012 presidential election. The homicide rate is correlated with violent crimes such as kidnapping, assault, and gang violence.

*Sex ratio* is the number of males per 100 females within the total population of a given municipality.

## Footnotes

1 In 2015 the country received 4.4% of the global total of international remittance inflows, and one-third of the Latin American total (Yearbook of Migration & Remittances, 2016, pp. 124, 127).

2 During Calderón’s presidency (2006–2012), the range of benefits covered by Oportunidades also expanded to include a food support program (“De Solidaridad a Prospera,” 2014).

3 The federal states of the country that receive the largest number of remittances are Michoacán (10%), Guanajuato (9.1%), Jalisco (8.9%), Mexico (6.3%), Puebla (5.5%), Oaxaca (5.2%), and Guerrero (5.1%). However the states whose economies are the most dependent on these transfers are Michoacán (9.9%), Guerrero (7.8%), Oaxaca (7.4%), and Zacatecas (6.8%) (Yearbook of Migration & Remittances, 2016, pp. 133–134).

4 While compelling, a recent study conducted by Imai et al. (2016) shows that there were no meaningful differences in registration rates, turnout rates, or electoral support for the incumbent in the 2000 presidential election across early- and late-participating villages. Similarly, using survey data from the Mexico 2000 Panel Study, Cornelius (2004) finds that CCT assistance had no impact on turnout rates in the same election.

5 Previous studies show that citizens are less likely to vote when they have not previously voted (Geys, 2006a, p. 26), have weak ties to political parties (Carreras & Castañeda-Angarita, 2013, pp. 6, 12, 18), have no (or low) faith in democratic institutions (Carlin, 2006; Seligson, 2002; Smith, 2009), think that the electoral process is marred by irregularities (Simpser, 2012; Carreras & Irepoglu, 2013; Carreras & Castañeda-Angarita, 2013), have low levels of interest in politics (Carreras & Castañeda-Angarita, 2013, p. 11), do not participate in activities that build social capital (Klesner, 2007; Carreras & Castañeda-Angarita, 2013, p. 14), and/or have not been victims of crime and violence (Trelles & Carreras, 2012).

6 According to existing research, older voters are more likely to vote than younger ones (Carreras & Castañeda-Angarita, 2013, pp. 10, 17). Since the relationship between age and turnout might be nonlinear, models include the squared term of this variable.

7 Various studies suggest that women are more likely to vote than men (Geys, 2006a, p.16; Camp, 2013, pp. 457–458). Also, cash transfers are often paid to women.

8 The idea here is that individuals with higher wealth and education levels should be better informed about politics, and in consequence more likely to be interested by, and participate in, elections (Brady et al., 1995; Carreras & Castañeda-Angarita, 2013, pp. 10, 17; Camp, 2013, p. 462)

9 Recent research in the context of Latin America shows that the unemployed are less likely to go to the polls (Carreras & Castañeda-Angarita, 2013, p. 11).

10 Various studies contend that interactions between parties and voters are more frequent and more personal in rural areas (Geys, 2006b, pp. 643–644). Thereby, voters who live in the countryside have better knowledge about politicians, and, consequently, more incentives for turning out at the polls (Carreras & Castañeda-Angarita, 2013, p. 14). Besides this, in Mexico CCT assistance and financial remittances are both biased toward rural areas.

11 If respondents intend to emigrate, they are less likely to be affected by politics in their home country, and therefore have fewer incentives to go to the polls (Geys, 2006b, p. 644).

12 “Within the class of matching estimators, using only a single match leads to the most credible inference with the least bias, at most sacrificing some precision” (Imbens, 2004, p. 14).

13 INEGI does not report the share of households that are beneficiary from the Oportunidades program in a given municipality. Nonetheless, greater spending on the program in a municipality equates to higher numbers of people benefiting from CCT assistance.

14 The weak instruments test proves that the instrument has a strong correlation with the endogenous explanatory variable, whereas the Wu-Hausman one identifies whether the instrumental variable estimation is as consistent as the OLS method.
